# New species of *Anthurium* Schott (Araceae) from the Kõkõi Eujã Natural Traditional Reserve of the Pacific coast, Cauca Department, Colombia

**DOI:** 10.3897/phytokeys.156.53303

**Published:** 2020-08-21

**Authors:** Jhon A. Sánchez-Taborda, Martin Llano-Almario, Alejandro Zuluaga-Tróchez

**Affiliations:** 1 Fundación Ecohabitats, Popayán, Colombia Fundación Ecohabitats Popayán Colombia; 2 Departamento de Biología, Universidad del Valle, Calle 13 # 100-00, Cali, Colombia Universidad del Valle Cali Colombia

**Keywords:** Eperãra Siapidaarã, *Phyllobates terribilis*, section *Digitinervium*, section *Tetraspermium*, Eperãra Siapidaarã, *Phyllobates terribilis*, sección *Digitinervium*, sección *Tetraspermium*

## Abstract

The Kõkõi Eujã Natural Traditional Reserve was created in 2019 to protect the golden poison frog (*Phyllobates terribilis* Myers, Daly & Malkin, 1978) in the Cauca Department of Colombia. As part of the biodiversity inventory of the reserve a new species of *Anthurium* with scandent habit was discovered. The new species is endemic to Colombia and it is more similar to *A. caldodsonii* Croat, *A. boekei* Croat, and *A. scandens* (Aubl.) Engl., but differs by having widely lanceolate leaves with acuminate apex, cuneate base, and acrodromous venation with three pairs of basal veins.

## Abstract (es)

La reserva natural tradicional Kõkõi Eujã fue creada en 2019 en el departamento de Cauca, Colombia, para proteger la rana dardo dorado (*Phyllobates terribilis* Myers, Daly & Malkin, 1978). Durante el proceso de realizar el inventario de la biodiversidad de la reserva se descubrió una especie nueva del género *Anthurium*. La especie nueva es endémica de Colombia, y es similar a *A. caldodsonii* Croat, *A. boekei* Croat y *A. scandens* (Aubl.) Engl., de las cuales se diferencia por tener hojas lanceoladas con ápice acuminado y base cuneada, y venación acródroma con tres pares de venas basales.

## Introduction

The genus *Anthurium* Schott has more than 1000 described species (WCSP 2020), but the estimated number is 2000 (Boyce and Croat 2018). Colombia is believed to be the country with the highest *Anthurium* diversity, and the Pacific slope of the Andes is the most diverse region in the country (Croat et al. 2010). This region overlaps with one of the world biodiversity hotspots, the Tumbes-Choco-Magdalena region (Myers et al. 2000), which remains poorly known, especially in the departments of Valle del Cauca, Cauca and Nariño, to the south of Colombia. The Pacific slope of the Andes region is not only important because of its biological diversity but also because of its cultural diversity. Several indigenous communities still survive in this part of Colombia, including the Eperãra Siapidaarã, which are known for the traditional use of the frog *Phyllobates terribilis* Myers, Daly & Malkin, 1978, the golden poison frog (Kõkõi in Sia language) as a source of poison used for hunting (Myers et al. 1978). However, the survival of these communities and the forests they inhabit is being threatened by factors like illegal mining, illegal timber extraction, and drug trafficking.

By initiative of four communities of the Eperãra Siapidaarã people belonging to the indigenous Calle Santa Rosa Reservation of the municipality of Timbiquí, in the Cauca Department, a new protected area was declared in order to protect the habitat of the golden poison frog. The new reserve was named the Kõkõi Eujã Natural Traditional Reserve and was included in the National Protected Areas System of Colombia (Paz et al. 2019). The process was led by the Ecohabitats Foundation and the Corporación Autónoma Regional del Cauca, with the support from the Rainforest Trust. The Calle Santa Rosa Indigenous Reservation is located between the municipalities of Timbiquí and López de Micay, and it covers about 21,320 ha. It is currently made up of four communities (La Sierpe, Calle Santa Rosa, Las Peñas and Unión Málaga) (Quiro 2017) with a population of 1,027 people in 234 families distributed in 115 homes. Its economy is based on fishing, and some crops, including bananas, cassava and coconut, in addition to the sugar cane cultivation to produce viche (an alcoholic beverage), which is sold in the municipal capital (Paz et al. 2019). The new reserve comprises about 11,641 ha corresponding to 56 percent of the total area of the reservation, the most representative land coverage is primary forest (11,412 ha), followed by secondary vegetation (144.9 ha), clean pastures (68.3 ha) and crops (15.9 ha).

## Methods

During 2018 the first author carried out field work off the Pacific coast in Timbiquí and López de Micay, in the Cauca Department of Colombia, to generate a baseline for the creation of the Kõkõi Eujã Natural Traditional Reserve. A new species of the genus *Anthurium* was found, and is described and illustrated here following Croat and Bunting (1979). All measurements are based on dried specimens.

## Taxonomic treatment

### Anthurium siapidaarae

Taxon classificationPlantaeAlismatalesAraceae

Zuluaga & Sánchez-Taborda sp. nov.

DF6C3D36-E135-53A0-96AE-5904369800FB

urn:lsid:ipni.org:names:77211167-1

Figs 1 , 2 , 3

#### Type.

Colombia. Cauca: municipio de Lopez de Micay, resguardo indígena Calle Santa Rosa, camino entre la orilla de la quebrada Bibango, afluente del río Saija, 02°57.467'N, 77°32.967'W y el bosque primario en la parte alta de la colina 02°58.056'N, 77°32.878'W, 16–65 m de altura, 10 September 2018, Jhon Alexander Sánchez-Taborda, Luis Alfonso Ortega, Carlos Robinson Quiro, José Tovar & Jainer Mejía 3141 (Holotype CUVC!).

**Figure 1.**
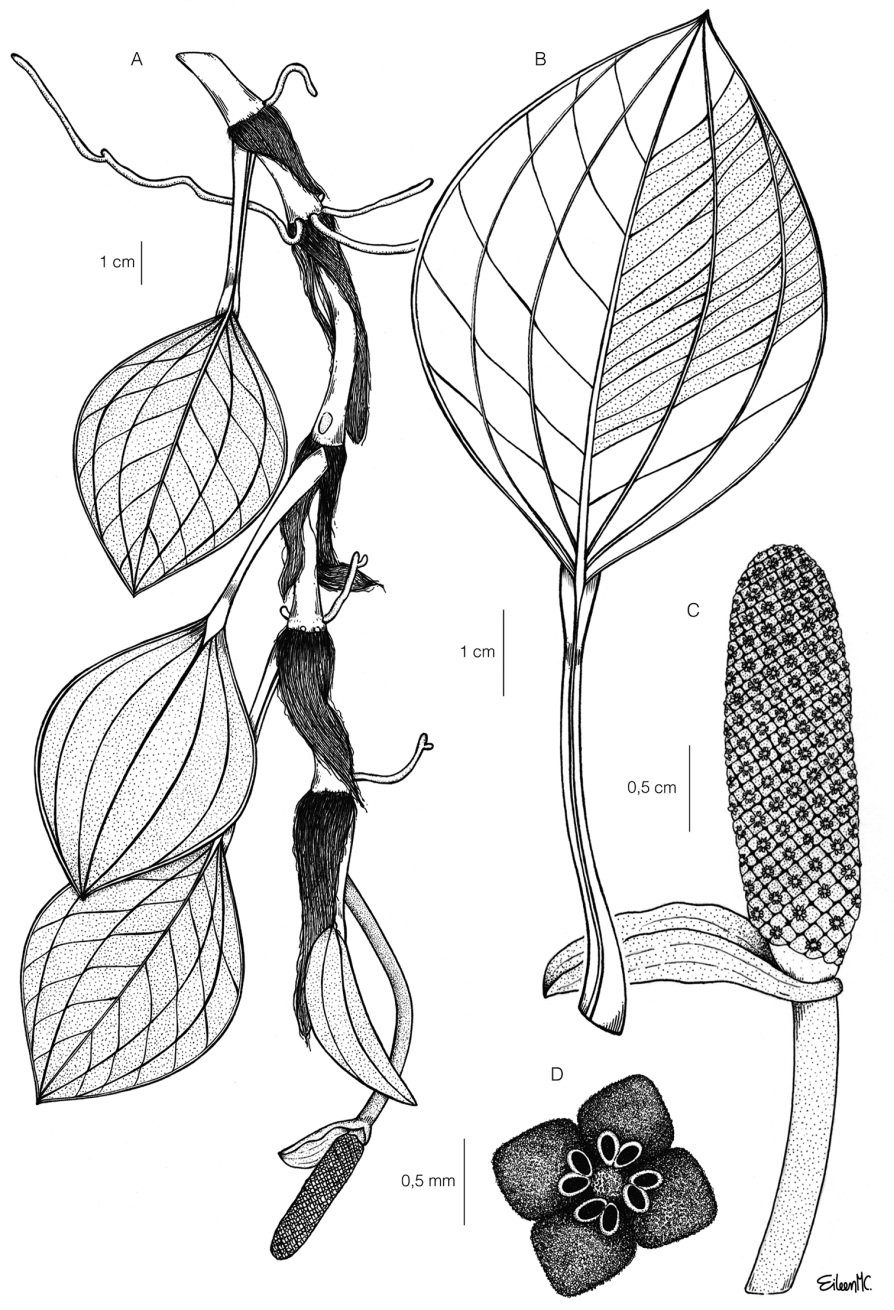
Illustration of *Anthurium siapidaarae* Zuluaga & Sánchez-Taborda, sp. nov. **A** habit and adult plant **B** leaf detail, adaxial view **C** inflorescence with spathe and peduncle **D** flower, frontal view. Illustration by Eillen Muñoz, based on the type collection J.A. Sánchez-Taborda 3141.

#### Diagnosis.

*A. siapidaarae* differs from *A. caldodsonii* Croat, *A. boekei* Croat and *A. scandens* (Aubl.) Engl. by having widely lanceolate leaves with acuminate apex, cuneate base, and acrodromous venation with three pairs of basal veins, one of them 0.3–1 mm from the margin (versus leaves acuminate apex, cuneate base, and acrodromous venation with two pairs of basal veins).

**Figure 2.**
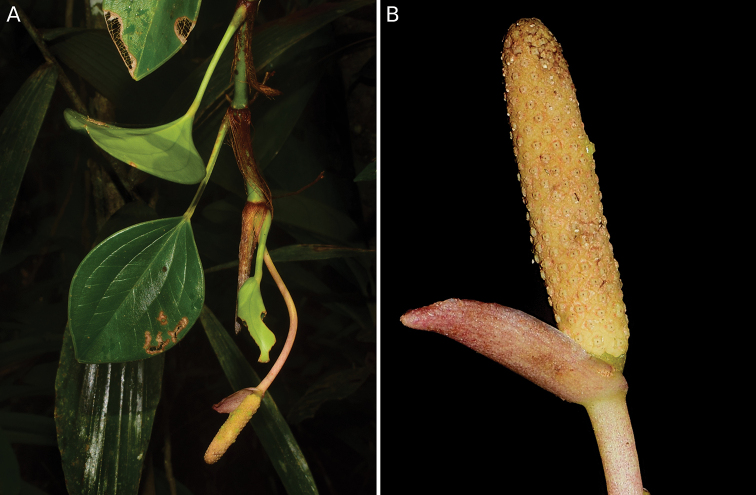
*Anthurium siapidaarae* Zuluaga & Sánchez-Taborda, sp. nov. **A** habit of living plant showing leaves and cataphylls **B** inflorescence. (Photographs by Jhon A. Sánchez-Taborda).

#### Description.

Epiphytic climbing herb; roots loose and spreading, slender, brown; stems cylindrical, green, drying green-brown; internodes 3–4.5 cm long, (2.7–) 3.7–4.4 mm diam when dry, weakly glossy, green, drying grayish-green; cataphylls 4–5.6 cm long, acuminate at apex, brown and glossy, drying light brown mate, persistent as fibers at the upper nodes, with only a few fibers remaining at basal nodes. ***Leaves*** scattered along stem; petioles ribbed canaliculate adaxially, (3.2-) 4.2–5.6 cm long, 0.2–0.3 mm diam, olive green, drying grayish-green; sheath 3–4 mm long, 2–4 mm width at midpoint, occupying less than ¼ the length of the petiole; geniculum 3–6 mm long, 3 mm diam, green, darker than the petiole and drying dark-brown; blades coriaceous, 4.6–7 cm long, 2.8–4.4 cm wide, 1.2–1.4 times longer than wide, widely lanceolate, acuminate at apex, cuneate at base, adaxial surface glossy dark-green, abaxial surface glossy light-green, black punctations present on both sides, margins slightly revolute; midrib impress and slightly paler above, prominent and dark green below; basal veins 2 per side when the plant is young, 3 per side when adult, one of them, the marginal collective vein, 0.3–1 mm from the margin; primary lateral veins 8–9 per side when the plant is young, 13 when adult, arising at an angle of 35–40° degree, concolorous and impress above, prominent and discolorous below. ***Inflorescence*** pendant; peduncle curved, cylindrical, slightly striate longitudinally, 8.5 cm long, 0.3 cm diam, 1.7 times longer than the petiole, reddish, weakly glossy, drying brown; spathe reddish and glossy, coming out at a 70° angle to the spadix, 1.7 cm long, 0.5 cm wide, lanceolate, with acuminate apex and decurrent base; stipe cylindrical ca. 2 mm long, 3 mm diam, green yellow, drying grayish-green; spadix light yellow, erect, cylindrical, 2.4 cm long, 0.4–0.5 cm diam, 1.4 times longer than spathe; ***flowers*** 5–6 in the principal spiral, 9–10 in the secondary spiral, outline rhombic in frontal view, ca 1.5 mm diam; tepals yellow to reddish, weakly glossy, drying dark-brown, 0.4 mm long, 0.7 mm wide when dry; pistil ca 1.3 mm diam, stigma capitate ca 0.4 mm diam with trichomes; stamens slightly exserted, filament short, anthers 0.3 mm wide; ***fruits*** not seen.

**Figure 3.**
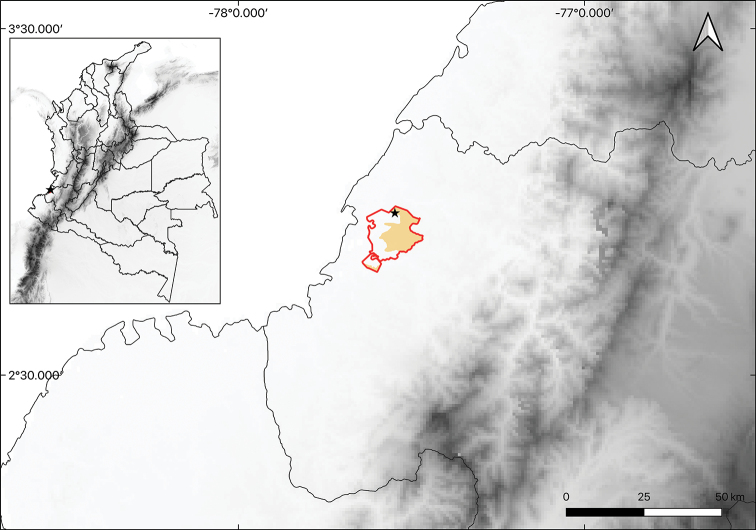
Distribution of *Anthurium siapidaarae* Zuluaga & Sánchez-Taborda, sp. nov. Boundaries of the Calle Santa Rosa Indigenous Reservation are shown in red, the area of the Kõkõi Eujã Natural Traditional Reserve in yellow. The star shows the locality where the species was collected.

#### Distribution and ecology.

*Anthurium siapidaarae* is endemic to Colombia, only known from the type locality in the municipality of López de Micay, Cauca. This species inhabits the Tropical Rain Forest between 30 and 100 m above sea level. *A. siapidaarae* is locally scarce, growing as an epiphyte inside dense forest with a closed canopy that exceeds 35 m in height, dominated by species of the genera *Protium* Burm. f., *Pouteria* Aubl., *Ficus* L., *Otoba* (A. DC.) H. Karst., *Ocotea* Aubl. and *Inga* Mill.

#### Phenology.

*Anthurium siapidaarae* was found flowering in September.

#### Etymology.

*Anthurium siapidaarae* is named after the indigenous community inhabiting the Calle Santa Rosa Indigenous Reservation. They belong to the Eperãra Siapidaarã people who live in the departments of Valle del Cauca, Cauca and Nariño in southwest Colombia. The word Siapidaara makes reference to the language Sia Pedeé spoken by these indigenous people.

#### Preliminary conservation status.

*Anthurium siapidaarae* is only known from one locality, where it is not abundant. Its populations are under protection thanks to the Kõkõi Eujã Natural Traditional Reserve, which has an area of 11641 ha (ca 115 km^2^). Despite being under protection, there are several pressures affecting the conservation of these forests, especially the increase of illegal crops surrounding the reservation and deforestation, which had a rate of 7.8 ha per year between 2001–2018 within the protected area (Paz et al. 2019). Under the IUCN criteria (IUCN 2017) we consider this species should be listed as Vulnerable.

#### Notes.

*Anthurium siapidaarae* could belong to sections *Tetraspermium* (Schott) Engl. Or Digitinervium Sodiro. The main character of section Tetraspermium is the presence of four seeds per fruit, so we cannot be completely sure of this placement due to the absence of fruits in the samples. There are also some similarities with species from section Digitinervium, mainly the thick leaves with glandular punctations, and three pairs of acrodromous veins. It is most similar to *A. caldodsonii*, *A. boekei*, endemic from Ecuador and *A. scandens*, a widespread species. All four species share the characters of section Tetraspermium, having scandent habit, long internodes, persistent fibrous cataphylls at least in the terminal nodes, and small leaves with a glandular-punctate lower surface. *A. siapidaarae* differs from the other species by having acrodromous venation with three pairs of basal veins, one of which, the collective marginal vein, is 0.3–1 mm from the margin (vs acrodromous venation with two pairs of basal veins in all other three species). It also differs from *A. caldodsonii* and *A. boekei*, only know from Ecuador, by having lanceolate leaves less than 1.4 times longer than wide, with acuminate apex and cuneate base (versus ovate to ovate-elliptical leaves, with truncate base and apex), and from *A. scandens*, by having a peduncle 8.5 cm long (vs. 1.5–6.5 cm long).

#### Additional specimens examined (paratypes).

Colombia. Cauca: municipio de López de Micay, resguardo indígena Calle Santa Rosa, camino entre la orilla de la quebrada Bibango, afluente del río Saija, 02°57.467'N, 77°32.967'W y el bosque primario en la parte alta de la colina 02°58.056'N, 77°32.878'W, 16–65 m de altura. 11 September 2018, Jhon Alexander Sánchez-Taborda, José Tovar & Jainer Mejía 3245 (CUVC).

## Supplementary Material

XML Treatment for Anthurium siapidaarae
